# Therapeutic Potential of *Luffa acutangula*: A Review on Its Traditional Uses, Phytochemistry, Pharmacology and Toxicological Aspects

**DOI:** 10.3389/fphar.2018.01177

**Published:** 2018-10-22

**Authors:** Parshuram Nivrutti Shendge, Sateesh Belemkar

**Affiliations:** Department of Pharmacology, School of Pharmacy and Technology Management, SVKM’s NMIMS, Dhule, India

**Keywords:** *Luffa acutangula*, traditional medicine, anthraquinones, saponin triterpene, antidiabetic activity, antioxidant activity

## Abstract

*Luffa acutangula* (Cucurbitaceae), a perennial plant grows mainly in India, Southeast Asia, China, Japan, Egypt, and other parts of Africa, it is widely used in the traditional Indian medicinal system to treat various health conditions. The plant has been used in jaundice, diabetes, hemorrhoids, dysentery, headache, ringworm infection, and leprosy. More than 50 chemical compounds have been isolated from a plant which mainly comprises flavonoids, anthraquinones, proteins, fatty acids, saponin triterpene, volatile components, and other phytoconstituents. Crude extract of plant and its isolated compounds possess broad pharmacological activities such as antidiabetic, hepatoprotective, antiulcer, anticancer, immunomodulatory, antihyperlipidemic, antioxidant, antimicrobial, CNS depressant, analgesic, and anti-inflammatory. The toxicological evaluation in preclinical studies reported safety of the plant for human consumption, but comprehensive evaluation in clinical studies is required. However, further investigation is necessary for transformation of experience based treatment of plant into evidence based information. Evaluation of pharmacological activity with indicative biomarkers will help to reveal the mechanism of action of chemical constituents of plant extract. The data from preclinical studies recommends clinical evaluation of safety and efficacy of the plant. The current paper summarizes up-to-date information about a review of the traditional uses, phytochemistry, pharmacological activities, and toxicology to highlight the future prospects of the plant.

## Introduction

Medicinal compounds from plant sources play a key role in prevention and treatment of disease since ancient time. Some of these compounds are toxic to plant predators, but have the beneficial effects in the treatment of human diseases. Demand for compounds isolated from plants is growing throughout the world and many pharma companies are currently conducting extensive research of these compounds in human health (Danish et al., 2011).

*Luffa acutangula* is a medicinal plant, usually referred as a ridge gourd. It is prevalent in subtropical region of Asia. India is considered as a primary center of origin. The plant is widely cultivated in India, Southeast Asia, China, Japan, Egypt, and other parts of Africa. Propagation of this plant is done through seeds and are sown in February–March or June–July (Munshi et al., 2010).

The purpose of the present review is to analyze the traditional uses, phytochemistry, pharmacological activity, and toxicological studies of plant. Moreover, the knowledge obtained from various experimental studies was critically assessed to provide justification for traditional and medicinal uses of *Luffa acutangula*.

## Botanical Aspects

*Luffa acutangula* (L.) Roxb. is classed in the Cucurbitaceae, a family of flowering of plants with 98 accepted genera and about 975 species. Many of the annual or perennial species native to temperate and tropical areas are fruit bearing or ornamental plants (Lucas et al., 2010; Encyclopaedia Britannica, 2018). The synonyms of plants are *Cucumis acutangulus*, *Cucurbita acutangula, Luffa foetida, Luffa drastica, Cucumis operculatus* Roxb., *Luffa gosa* Ham. (Quattrocchi, 2012).

The plant has different names in different languages of India such as: English: Ridged gourd, angled loofah, ribbed gourd, Chinese okra, silk squash (En); Hindi: Turai, Kadaviturai; Marathi: Dodaka; Sanskrit: Dhamargava, Koshataki; Bengali: Titotorai, Titojhinga, Titodhundal, Jhinga, Ghoshalata; Kannada: Kahire, Kahi heere, Naaga daali balli; Malayalam: Athanga; Tamil: Itukari, Itukarikkoti, Kacappi, Kacappuppirkku, Kaccam, Kaippuppirkku, Karniti; Telugu: Adavibira, Chedubira, Sendubirai, Adavi beera, Chathi beera (Nadkarni, 1996).

The roots of the plant are yellowish brown in color and cylindrical in shape. Longitudinal wrinkles on root contribute to their rough texture. Five angled, glabrous stem is brownish yellow in color along with tendrils up to 6-fid. Flowers are regular, unisexual and consists of yellow petals. Female flowers are yellow colored solitary, 2–15 cm long on pedicels, with inferior, longitudinally ridged ovary and 3-lobed stigma while male flowers are light greenish in color, consist of three free stamens with yellow corolla inserted into the receptacle tube. Leaves are simple, alternate and orbicular in outline with 15–20 cm long, palmately 5–7 angled, triangular to broadly rounded lobes and pale green in color. Veins and vein islets are prominent. Fruits are cylindrical, pale yellowish-brown in color, bitter in taste, tapered toward the base and are covered with 8–10 prominent ribs. Inner part of the fruit is three chambered, fibrous and easily detachable from the outer part. Seeds are elliptical and black colored (Basu and Kirtikar, 1987).

## Traditional Uses and Ethnopharmacology

Different parts of *Luffa acutangula* have been used extensively by different ethnic groups in India for medicinal purposes. In Maharashtra and the tribal areas of Madhya Pradesh, leaves and fruit powder are used for the treatment of jaundice (Das and Basu, 1997; Samvatsar and Diwanji, 2000).

A local inhabitant from reserve forest of Mahadevpur (previously in Andhra Pradesh now in Telangana) widely uses the fruit for diabetes treatment (Kanaka et al., 2013). Apart from this, the plant is also used by the tribes of western Maharashtra on insect bite. Fruit powder is applied topically to treat swollen hemorrhoids. The kernel of the seed is used as an efficient remedy for dysentery while the juice of the fruit is applied to cure a headache (Dandge et al., 2010). Oral administration of seed powder is extensively used for the treatment of urinary bladder stone in Rajasthan (Katewa et al., 2004). Local application of pulverized leaves is reported to be useful in splenitis, hemorrhoids, ringworm infection, and leprosy while the juice of the leaves is administered into the eye for treatment of granular conjunctivitis in children (Mahbubar, 2013). In addition, the fruit possesses demulcent and diuretic properties while the seeds have purgative, emetic and anthelmintic properties. The dried fruit powder is useful in preventing premature graying of hair. The root of the plant is laxative and used in dropsy (Nadkarni, 1996).

## Phytochemistry

The phytochemical studies have resulted in isolation and identification of approximately 50 compounds, such as flavonoids, anthraquinones, proteins, fatty acids, saponin triterpene, volatile components, and other phytoconstituents (Table 1).

**Table 1:** Isolated phytochemicals of *Luffa acutangula.*

Compound no.	Compound	Extract	Part	Type	Reference
1	Luffaculin 1	SDS-PAGE	Seed	Protein	Junkai et al., 2002
2	Luffaculin 2	SDS-PAGE	Seed	Protein	Junkai et al., 2002
3	Luffangulin	–	Seed	Protein	Wang and Ng, 2002
4	Apigenin-7-glucoside	–	Leaf and flower	Flavonoids	Schilling and Heiser, 1981
5	Luteolin-7-glucoside	–	Leaf and flower	Flavonoids	Schilling and Heiser, 1981
6	1,8-dihydroxy-4-methylanthracene-9,10-dione	Ethanolic extract	Aerial parts	Anthraquinone	Vanajothi and Srinivasan, 2015
7	Myristic acid	Oil	Seed	Fatty acid	Kamel and Blackman, 1982
8	Palmitic acid	Oil	Seed	Fatty acid	Kamel and Blackman, 1982
9	Stearic acid	Oil	Seed	Fatty acid	Kamel and Blackman, 1982
10	Oleic acid	Oil	Seed	Fatty acid	Kamel and Blackman, 1982
11	Linoleic acid	Oil	Seed	Fatty acid	Kamel and Blackman, 1982
12	Oleanolic acid 3-*O*-β-D-glucopyranosyl-(1→2)-β-D-glucopyranoside (Acutoside-A)	Methanolic extract	Aerial parts	Oleanane-type triterpene	Nagao et al., 1991
13	28-*O*-[*O*-β-D-xylopyranosyl-(1→4)-*O*-α-L-rhamnopyranosyl-(1→2)-α-L-arabinopyranosyl] ester (Acutoside-B)	Methanolic extract	Aerial parts	Oleanane-type triterpene	Nagao et al., 1991
14	28-*O*-[*O*-β-D-xylopyranosyl-(1→3)-*O*-β-D-xylopyranosyl-(1→4)-*O*-α-L-rhamnopyranosyl-(1→2)-α-L-arabinopyranosyl] ester (Acutoside-D)	Methanolic extract	Aerial parts	Oleanane-type triterpene	Nagao et al., 1991
15	28-*O*-[*O*-α-L-arabinopyranosyl-(1→3)-*O*-β-D-xylopyranosyl-(1→4)-*O*-α-L-rhamnopyranosyl-(1→2)-α-L-arabinopyranosyl] ester (Acutoside-E)	Methanolic extract	Aerial parts	Oleanane-type triterpene	Nagao et al., 1991
16	28-*O*-[*O*-β-D-xylopyranosyl-(1→3)-[*O*-β-D-xylopyranosyl-(1→4)-*O*-α-L-rhamnopyranosyl-(1→2)-α-L-arabinopyranosyl] ester (Acutoside-F)	Methanolic extract	Aerial parts	Oleanane-type triterpene	Nagao et al., 1991
17	28-*O*-β-D-xylopyranosyl-(1→3)-[*O*-α-L-arabinopyranosyl-(1→3)-*O*-β-D-xylopyranosyl-(1→4)]-*O*-α-L-rhamnopyranosyl-(1→2)-α-L-arabinopyranosyl] ester (Acutoside-G)	Methanolic extract	Aerial parts	Oleanane-type triterpene	Nagao et al., 1991
18	Machaelinic acid (Acutoside-C)	Methanolic extract	Aerial parts	Oleanane-type triterpene	Nagao et al., 1991
19	3-Methyl-1-butanol	SPME coupled with GC-MS	Flower	Volatile components	Fernando and Grun, 2001
20	4,5-Dimethyl-1-hexene	SPME coupled with GC-MS	Flower	Volatile components	Fernando and Grun, 2001
21	α-Thujene	SPME coupled with GC-MS	Flower	Volatile components	Fernando and Grun, 2001
22	α-Pinene	SPME coupled with GC-MS	Flower	Volatile components	Fernando and Grun, 2001
23	Sabinene	SPME coupled with GC-MS	Flower	Volatile components	Fernando and Grun, 2001
24	β-Pinene	SPME coupled with GC-MS	Flower	Volatile components	Fernando and Grun, 2001
25	β-Myrcene	SPME coupled with GC-MS	Flower	Volatile components	Fernando and Grun, 2001
26	D,L-Limonene	SPME coupled with GC-MS	Flower	Volatile components	Fernando and Grun, 2001
27	1,8-Cineole	SPME coupled with GC-MS	Flower	Volatile components	Fernando and Grun, 2001
28	β-Ocimene (Z)	SPME coupled with GC-MS	Flower	Volatile components	Fernando and Grun, 2001
29	β-Ocimene (E)	SPME coupled with GC-MS	Flower	Volatile components	Fernando and Grun, 2001
30	β-Terpinene	SPME coupled with GC-MS	Flower	Volatile components	Fernando and Grun, 2001
31	γ-Terpinene	SPME coupled with GC-MS	Flower	Volatile components	Fernando and Grun, 2001
32	Methyl, methyl ethyl substituted benzene	SPME coupled with GC-MS	Flower	Volatile components	Fernando and Grun, 2001
33	*trans*-Linalool oxide	SPME coupled with GC-MS	Flower	Volatile components	Fernando and Grun, 2001
34	*trans*-Dihydrocarvone	SPME coupled with GC-MS	Flower	Volatile components	Fernando and Grun, 2001
35	Linalool	SPME coupled with GC-MS	Flower	Volatile components	Fernando and Grun, 2001
36	*cis*-Sabinene hydrate	SPME coupled with GC-MS	Flower	Volatile components	Fernando and Grun, 2001
37	α-Thujone	SPME coupled with GC-MS	Flower	Volatile components	Fernando and Grun, 2001
38	2-methyl-6-methylene-1,7-octadien-3-one	SPME coupled with GC-MS	Flower	Volatile components	Fernando and Grun, 2001
39	3,4-dimethyl-2,4,6-octatriene	SPME coupled with GC-MS	Flower	Volatile components	Fernando and Grun, 2001
40	Epoxylinelol	SPME coupled with GC-MS	Flower	Volatile components	Fernando and Grun, 2001
41	α-Terpineol	SPME coupled with GC-MS	Flower	Volatile components	Fernando and Grun, 2001
42	1H-Indole	SPME coupled with GC-MS	Flower	Volatile components	Fernando and Grun, 2001
43	Neryl acetate	SPME coupled with GC-MS	Flower	Volatile components	Fernando and Grun, 2001
44	2,3-dihydro,3,5-dihydroxy-6-methyl-(4H)-pyran-4-one	Ethanolic extract	Fruit	Other	Suryanti et al., 2017
45	3,7,11,15-tetramethyl-2-hexadecen-1-ol	Ethanolic extract	Fruit	Other	Suryanti et al., 2017
46	(3β, 20R)-cholest-5-en-3-ol	Ethanolic extract	Fruit	Other	Suryanti et al., 2017
47	9,12,15-octadecatrienoic acid methyl ester	Ethanolic extract	Fruit	Other	Suryanti et al., 2017
48	Citronellyl tiglate	Ethanolic extract	Fruit	Other	Suryanti et al., 2017
49	Ascorbic acid	Ethanolic extract	Fruit	Other	Nagarajaiah and Prakash, 2014
50	Carotene	Ethanolic extract	Fruit	Other	Nagarajaiah and Prakash, 2014

**Table 2:** Pharmacological activity of extracts and fractions of *Luffa acutangula.*

Activity	Part used	Extract/Compound/Dose	Animal/Cell lines/Bacterials	Model/Diseases	Results	Reference
**Hepatoprotective activity**	Fruit	Ethanolic (150 mg/kg, p.o.) and petroleum ether (150 mg/kg, p.o.) extract	Albino rat	Carbon tetrachloride induced liver necrosis	SGPT, SGOT, serum alkaline phosphatase (ALP), serum bilirubin, serum cholesterol, triglycerides, serum high density, lipoproteins (SHDL), serum total proteins and serum albumin levels were reduced by alcoholic extract	Ibrahim et al., 2014
	Fruit	Hydro-alcoholic (70%) extract; 100, 200, and 400 mg/kg p.o.	Wistar rat	Carbon tetrachloride and rifampicin induced hepatotoxicity	Significantly reduced serum marker enzyme (AST, ALP, ALT, and LDH) levels; non-enzymatic and enzymatic antioxidant (glutathione, catalase, and superoxide dismutase) levels were increased	Jadhav et al., 2010
	Fruit	Alcoholic extract further partitioned with toluene, chloroform, ethyl acetate; 100 mg/kg p.o.	Albino rat	Paracetamol induced hepatotoxicity	Significantly increased direct bilirubin level while ALT, AST, and ALP levels were restored to normal an ethyl acetate fraction of alcoholic extract	Mishra and Mukerjee, 2017
	Leaves	Ethanolic extract; 200, 400, 600 mg/kg p.o.	Wistar rat	Carbon tetrachloride induced	Elevated levels of serum markers (SGPT, SGOT, ALP) reduced and significantly improved levels of glutathione peroxidase, glutathione-*S*-transferase, reduced glutathione, superoxide dismutase, catalase, and lipid peroxidation by leaf extract	Ulaganathan et al., 2010
**Antidiabetic activity**	Fruit	Ethanolic extract (95%); 200 mg/kg i.p.	Long Evans female rat	Alloxan monohydrate induced	Extract reduced glucose level by 51.10%; reduced glycogen content of diabetic rat was attenuated by treatment with extract	Sharmin et al., 2013
	Fruit	Methanolic extract; 50, 100, 200, and 400 mg/kg p.o.	Swiss albino mice	Single dose of glucose (2 g/kg of body weight)	Glucose levels decreased in dose dependant manner	Juma et al., 2013
	Fruit	Lyophilized ethanolic extract (50%); 200 and 400 mg/kg p.o.	Diabetic Wistar rat	Streptozotocin induced	Blood glucose level was significantly reduced in dose dependent manner. Biochemical estimation of serum indicated decreased levels of SGPT, SGOT and ALP	Mohan Raj et al., 2012
	Fruit	Aqueous and methanolic extract; 200 and 400 mg/kg p.o.	Swiss albino mice	Streptozotocin induced	Methanolic extract of *Luffa acutangula* fruit decreased levels of fasting serum glucose, glycosylated Hb, ALT, and AST while increased level of liver glycogen which contribute to attenuate hyperglycemic condition in mice	Pimple et al., 2011
	Fruit	Ether, chloroform, ethanol, and aqueous extracts; 200 mg/kg p.o.	Wister rats	Alloxan induced	Chloroform and alcoholic extracts of fruits of *Luffa acutangula* shown significant (*p* < 0.01) blood glucose reduction	Patil et al., 2010
	Leaves	Methanol extract	Swiss Webster mice	Glucose solution at the concentration of 0.010 ml/g	Significant glucose lowering activity in oral glucose tolerance test	Quanico et al., 2008
**Antihyperlipidemic activity**	Fruit	Hydro-alcoholic extract	Wistar rat	Alloxan induced	Significant reduction in glucose	Singh et al., 2014
	Fruit	Ethanolic extract (95%)	Long Evans female rat	Alloxan induced	Reduced levels of total cholesterol (TC), triglyceride (TG), and low-density lipoprotein (LDL) by 38.38, 79.64, and 85.66%, respectively	Sharmin et al., 2013
	Fruit	Aqueous and methanolic extract; 200 and 400 mg/kg p.o.	Mice	Streptozotocin induced	Extract significantly (*P* < 0.05) increased levels of high density lipoprotein and reduced serum total cholesterol, triglycerides, low density lipoprotein, very low density lipoprotein	Pimple et al., 2011
**Anticancer activity**	Fruit	Methanolic and aqueous extract; 200 and 400 mg/kg, oral	Swiss albino mice	Dalton’s Lymphoma Ascites (DLA) cell induced	Methanolic and aqueous extract significantly reduced development of solid tumor in mice	Dashora and Chauhan, 2015
		Ethanolic extract	Human lung cancer cell line (NCl-H460).	–	The IC_50_ value of *Luffa acutangula* extract was found to be 20 μg/ml in MTT assay	Vanajothi et al., 2012
**Analgesic and anti-inflammatory activity**	Leaves	Ethyl acetate and ethanol extracts; 250 and 500 mg/kg	–	Carrageenan induced hind paw edema and cotton pellet granuloma models	Ethanolic extract showed significant activity then ethyl acetate extract.	Iyyamperumal et al., 2013
	Seed	Ethanolic extract; 100, 200, and 300 mg/kg, oral	Albino rat	Carrageenan induced paw edema; tail flick and tail immersion method	The ethanolic extract showed significant anti-inflammatory activity at the dose of 300 mg/kg while analgesic activity at the dose of 400 mg/kg	Gill et al., 2011
**Antibacterial activity**	Leaves	Silver nanoparticles prepared from aqueous extract	*Escherichia coli*, *Klebsiella pneumonia*, *Proteus vulgaris*, *Pseudomonas aeruginosa*, *Salmonella paratyphi* and *Staphylococcus aureus*	–	The Minimum Inhibitory Concentration (MIC) values showed that silver nanoparticle of leaves extract was more effective against Gram-positive bacteria at lower concentration then Gram-negative bacteria	Moideen and Prabha, 2014
	Aerial parts	Methanolic extract; 10, 25, and 50 mg/ml	Earthworms	–	The dose of 50 mg/ml exhibited good anthelmintic activity with paralysis time nearly about 24 min and death time about 45 min	Rahman et al., 2014
	Fruit, seed, leaves, and root	Methanolic and aqueous extracts	*E. coli*, *Staphylococcus aureus*, *Klebsiella pneumonia, Fusarium* sp., *Aspergillus niger*	–	Antimicrobial activity of different parts was solvent dependent	Jadhav and Chavan, 2013
	Fruit and leaves	Aqueous extract	*E. coli*, *Staphylococcus aureus* and *Pseudomonas aeruginosa* species	–	Fruit extract exhibit more potent antibacterial and antifungal activity then leaf extract	Dandge et al., 2012
	Leaves	*n*-hexane, chloroform and ethyl acetate extracts	–	Disk diffusion method	Activity shown as: *n*-hexane > chloroform extract > ethyl acetate	Bulbul et al., 2011
**Immunomodulatory activity**	Fruit pericarp	Ethanolic extract; 100 and 200 mg/kg, p.o.	Swiss albino mice	*In vivo* phagocytic assay and neutrophil adhesion assay	Increased phagocytosis and % neutrophil adhesion assay was observed with dose of 200 mg/kg	Kalasakar and Surana, 2014
**CNS depressant activity**	Fruit	Ethanolic extract; 5 and 10 mg/kg, p.o.	Swiss mice	Behavioral changes, exploratory activity, barbiturates sleeping time animal models	CNS depressant activity of extract is dose dependant activity	Misar et al., 2004
**Antiulcer activity**	Dried fruit pulp extract	Methanolic and aqueous; 200 mg/kg p.o.	Rat	Ulcer in diabetic rat was induced by aspirin	Methanolic extract exhibit dose dependent glucose lowering and mucosal defensive action	Pimple et al., 2012

### Proteins

Various ribosome inactivating proteins (RIPs) were isolated from different parts of *Luffa acutangula*. Medicinal applications of RIP have received wide attention, as they possess various pharmacological activities including abortifacient (Jin, 1985), antifungal (Leah et al., 1991), anti-tumor (Bolognesi and Polito, 2004), antivirus and HIV-1 integrase inhibitory activities (Au et al., 2000; Wang et al., 2002).

Junkai et al. (2002) isolated two RIPs, luffaculin 1 **(1)** and 2 **(2)** from seeds by using sodium dodecyl sulfate-polyacrylamide gel electrophoresis (SDS-PAGE). The molecular mass of luffaculin 1 and 2 was found to be at 28 kD. Significant anticancer activity was shown by both RIP in human leukemia K562 cells with an IC_50_ value of 1.1 × 10^−6^ and 2.0 × 10^°7^ mol/L, respectively (Junkai et al., 2002).

Another RIP, luffangulin **(3)** was isolated from seed which inhibited cell-free translation (IC_50_ = 3.5 nM) but showed no activity against HIV-1 reverse transcriptase (Wang and Ng, 2002).

### Flavonoids

**FIGURE 1:**
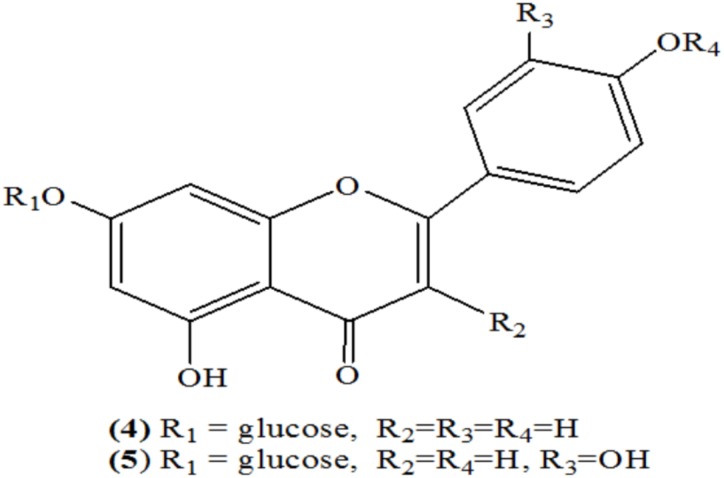
Flavonoids.

Schilling and Heiser (1981) isolated total 10 flavonoids from different species of Luffa. Among these, only two flavonoids, i.e., apigenin-7-glucoside (4) and luteolin-7-glucoside (5) were present in leaf and flower (Figure 1) (Schilling and Heiser, 1981).

### Anthraquinone

**FIGURE 2:**
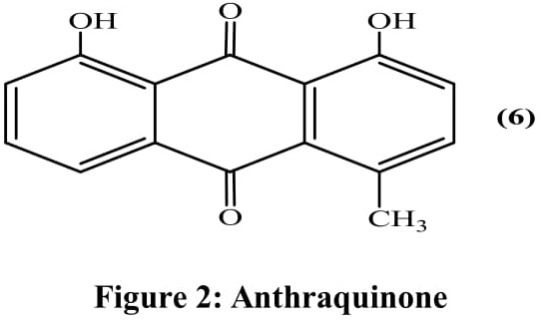
Anthraquinone.

Anthraquinone derivative 1,8-dihydroxy-4-methylanthracene-9,10-dione **(6)** was isolated using bioassay-guided approach from the ethanolic extract of aerial parts (Figure 2). Only five fractions out of the fourteen were evaluated for anti-cancer activity against non-small cell lung cancer cells (NCI-H460). Fraction obtained at second position significantly decreased growth of cell with IC_50_ value of 10 mg/ml concentration (Vanajothi and Srinivasan, 2015).

### Fatty Acids

**FIGURE 3:**
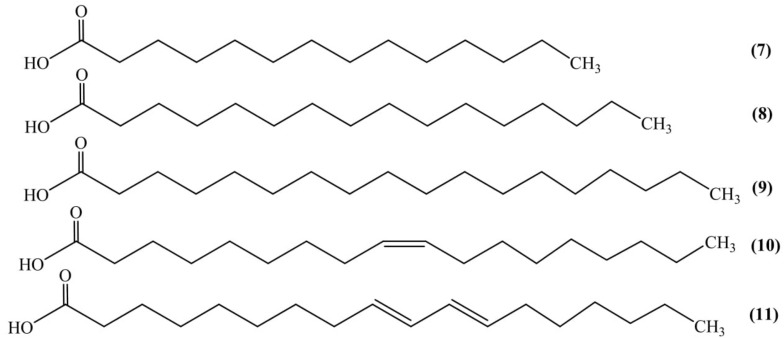
Fatty acids.

Nutritional evaluation of seed showed the presence of fats, proteins, and minerals. The protein and fat obtained from the kernel were 39% and 44% of the total weight, respectively. Investigational analysis of seed oil showed the presence of total saturated (32.1%) and unsaturated (67.9%) fatty acids which were recognized as myristic (0.45%) **(7)**, palmitic (20.9%) **(8)**, stearic (10.8%) **(9)**, oleic (24.1%) **(10)**, and linoleic (43.7%) acid **(11)** (Figure 3). Iodine value, saponification value and acid value of the seed oil were found to be 99.5, 190.8, and 10.5, respectively. The minerals like Fe, Ca, Zn, Cu, P, and Mg were also identified from the seed kernel (Kamel and Blackman, 1982).

### Saponin Triterpene

**FIGURE 4:**
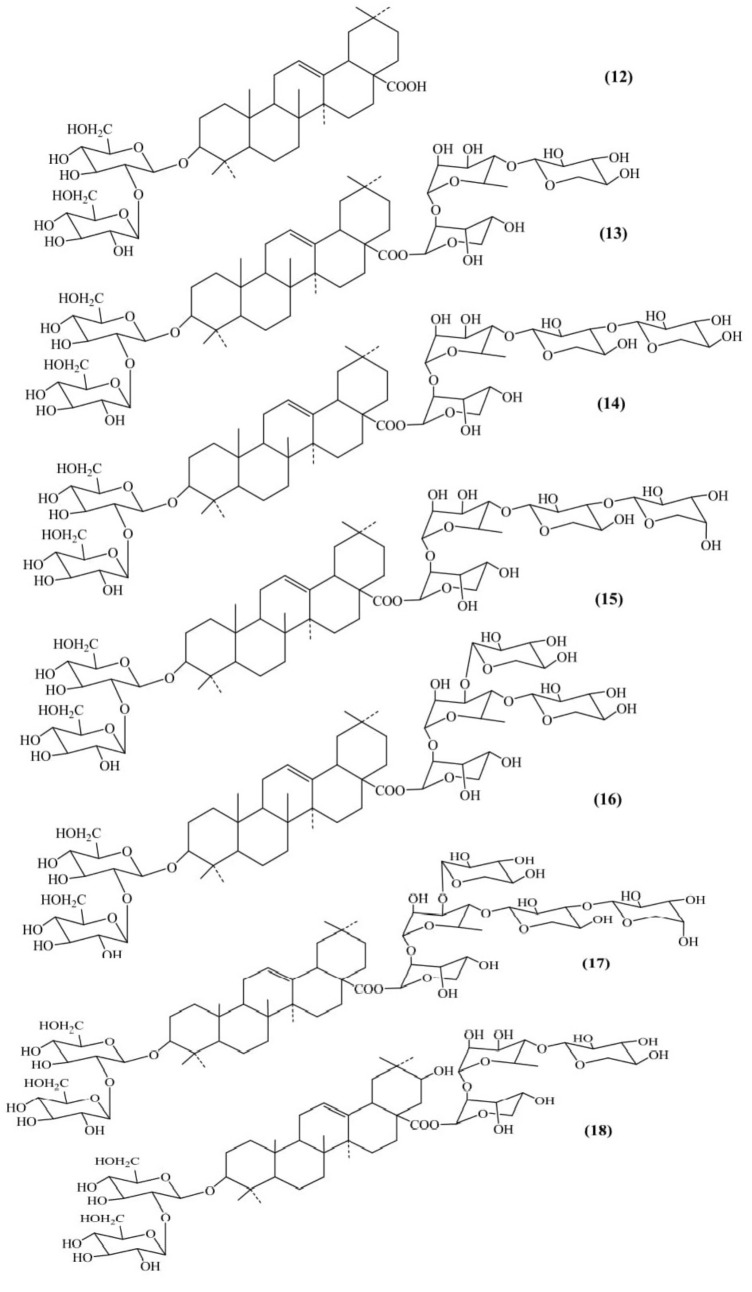
Saponin triterpene.

Seven saponins belonging to the oleanane – type triterpene were isolated from the aerial parts of *Luffa acutangula* (Figure 4). Methanolic extract was repeatedly chromatographed on normal and reversed phase to obtain designated Acutosides A to G. The triterpene saponins isolated were named as: oleanolic acid 3-*O*-β-D-glucopyranosyl-(1→2)-β-D-glucopyranoside (Acutoside-A) **(12)**, 28-*O*-[*O*-β-D-xylopyranosyl-(1→4)-*O*-α-L-rhamnopyranosyl-(1→2)-α-L-ara- binopyranosyl] ester (Acutoside-B) **(13)**, 28-*O*-[*O*-β-D-xylopyranosyl-(1→3)-*O*-β-D-xylopyranosyl-(1→4)-*O*-α-L-rha-mnopyranosyl-(1→2)-α-L-arabinopyranosyl] ester (Acuto-side-D) **(14)**, 28-*O*-[*O*-α-L-arabinopyranosyl-(1→3)-*O*-β-D-xylopyranosyl-(1→4)-*O*-α-L-rhamnopyranosyl-(1→2)-α-L-ara-binopyranosyl] ester (Acutoside-E) **(15)**, 28-*O*-[*O*-β-D-xylopyranosyl-(1→3)-[*O*-β-D-xylopyranosyl-(1→4)-*O*-α-L-rha-mnopyranosyl-(1→2)-α-L-arabinopyranosyl] ester (Acuto-side-F) **(16)**, 28-*O*-β-D-xylopyranosyl-(1→3)-[*O*-α-L-arabino- pyranosyl-(1→3)-*O*-β-D-xylopyranosyl-(1→4)]-*O*-α-L-rham-nopyranosyl-(1→2)-α-L-arabinopyranosyl] ester (Acutoside-G) **(17)**. Acutoside-C (machaelinic acid, = 21-β-hydroxyoleanolic acid) **(18)** contains the same sugar moiety as that of Acutoside-B. The study revealed that Acutosides have a common prosapogenin structure but differ in the ester-linked sugar moieties structure (Nagao et al., 1991).

### Volatile Components

**FIGURE 5:**
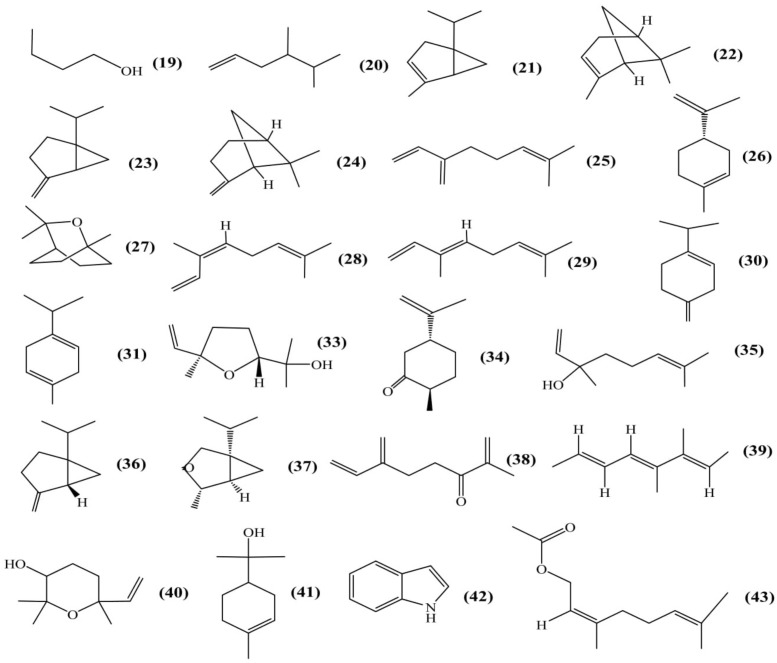
Volatile components.

Total 25 volatile components from flower were isolated using solid-phase microextraction (SPME) coupled with capillary gas chromatography/mass spectrometry (GC–MS) (Figure 5). Out of 25 compounds, 16 volatiles were positively identified and 9 were tentatively identified as 3-methyl-1-butanol **(19)**; 4,5-dimethyl-1-hexene **(20)**; α-thujene **(21)**; α-pinene **(22)**; sabinene **(23)**; β-pinene **(24)**; β-myrcene **(25)**; D,L-limonene **(26)**; 1,8-cineole **(27)**; β-ocimene (Z) **(28)**; β-ocimene (E) **(29)**; β-terpinene **(30)**; γ-terpinene **(31)**; methyl, methyl ethyl substituted benzene **(32)**; *trans*-linalool oxide **(33)**; *trans*-dihydrocarvone **(34)**; linalool **(35)**; *cis*-sabinene hydrate **(36)**; α-thujone **(37)**; 2-methyl-6-methylene-1,7-octadien-3-one **(38)**; 3,4-dimethyl-2,4,6-octatriene **(39)**; epoxylinelol **(40)**; α-terpineol **(41)**; 1H-indole **(42)**; neryl acetate **(43)** (Fernando and Grun, 2001).

### Other Phytoconstituents

**FIGURE 6:**
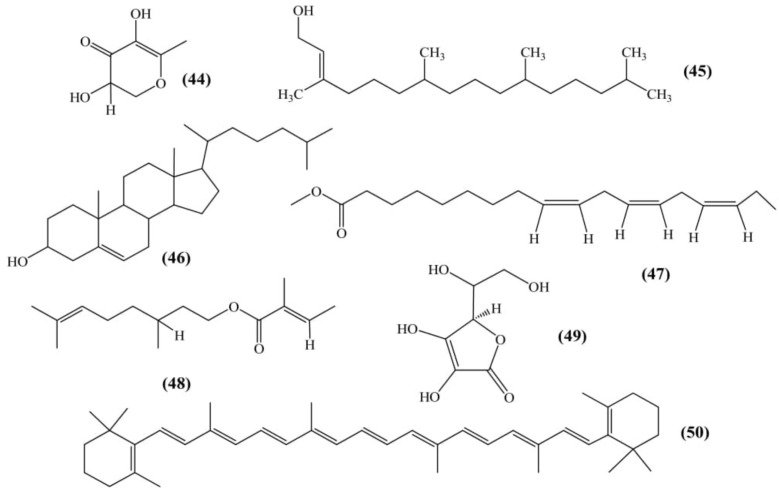
Other phytoconstituents.

Six compounds were isolated and analyzed from ethanolic fruit extract using GC-MS named as: 2,3-dihydro,3,5-dihydroxy-6-methyl-(4H)-pyran-4-one **(44)**; 3,7,11,15-tetramethyl-2-hexadecen-1-ol **(45)**; (3β, 20R)-cholest-5-en-3-ol **(46)**; *n*-hexadecanoic acid **(08)**; 9,12,15-octadecatrienoic acid methyl ester **(47)** and citronellyl tiglate **(48)** (Figure 6) (Suryanti et al., 2017).

Nagarajaiah and Prakash (2014) evaluated physical characteristics and the chemical composition of the dehydrated fruit peel. The results exhibited the presence of iron (4.74 mg), calcium (416 mg), phosphorous (233 mg), ascorbic acid (35 mg) **(49)**, carotene (36.96 mg) **(50)** and tannin (778.20 mg) per 100 gm of peel (Figure 6) (Nagarajaiah and Prakash, 2014).

## Pharmacological Activity of *Luffa acutangula*

**FIGURE 7:**
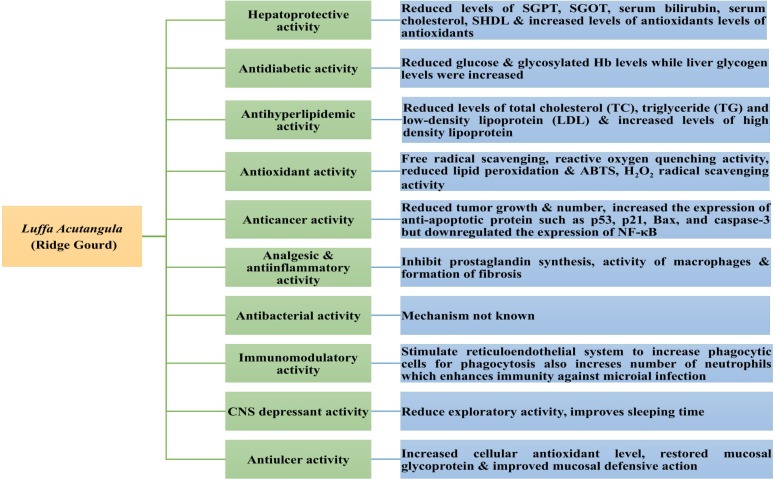
Possible mechanisms for pharmacological activity.

The extracts and purified compounds from *Luffa acutangula* have been investigated for various pharmacological activities using *in vitro* and *in vivo* models (Table 2 and Figure 7). Extracts from different parts of the plant exhibited potent hepatoprotective, antidiabetic, antihyperlipidemic, antioxidant, anticancer, antibacterial, CNS depressant, immunomodulatory, and antiulcer activity. Some of these activities are discussed below.

### Hepatoprotective Activity

Various studies have reported therapeutic potential of *Luffa acutangula* against liver diseases. Ethanolic fruit extract showed significant hepatoprotective activity compared to pet ether extract in carbon tetrachloride-induced liver necrosis. It also significantly reduced SGPT, SGOT, serum alkaline phosphatase (ALP), serum bilirubin, serum cholesterol, triglyceride (TG), serum high density lipoproteins (SHDLs), serum total proteins and serum albumin. Histopathological studies of liver showed early necrosis in petroleum ether extract while no necrosis was observed in the ethanolic extract, indicating the hepatoprotective potential of the latter (Ibrahim et al., 2014).

In another study, researchers investigated hepatoprotective activity of hydro-alcoholic (70%) fruit extract against carbon tetrachloride and rifampicin-induced hepatotoxicity in Wistar rats. The doses of 100, 200, and 400 mg/kg, p.o. significantly reduced serum marker enzyme (AST, ALP, ALT, and LDH) levels which attributed to the hepatoprotective action of the extract in the rat (Jadhav et al., 2010).

Hepatoprotective activity of different fractions of alcoholic fruit extract was evaluated by Mishra and Mukerjee (2017) against paracetamol induced liver toxicity. Toluene, chloroform, and ethyl acetate fractions of ethanolic extract were administered orally (100 mg/kg) and biochemical parameters were measured. Ethyl acetate fraction increased direct bilirubin level while ALT, AST, and ALP levels were restored to normal when compared with other fractions. Histopathological evaluation of live cells indicated the absence of necrosis with less vacuole formation (Mishra and Mukerjee, 2017).

Furthermore, Ulaganathan et al. (2010) screened hepatoprotective activity of ethanolic extract of the leaves against carbon tetrachloride. Carbon tetrachloride induced elevated levels of serum markers (SGPT, SGOT, and ALP) were brought to normal by oral administration of leaf extract. Tissue specific antioxidant activity of extract have been observed with the help of improved levels of glutathione peroxidase, glutathione-*s*-transferase, reduced glutathione, superoxide dismutase, catalase, and lipid peroxidation (Ulaganathan et al., 2010).

Taken together, these results support the traditional use of *Luffa acutangula* as hepatoprotective agent. However, hepatoprotective effect is still unconvincing as humans studies were not performed. Hence, ridge gourd is worth considering for treatment of hepatic diseases in human and therefore, should be extensively studied.

### Antidiabetic Activity

Ancient literature reported the use of fruit juice in an adrenal variety of diabetes (Nadkarni, 1996). Various studies have been performed to prove antidiabetic effect of the plant. Hypoglycemic activity of ethanolic extract (95%) of *Cucumis sativus*, *Lagenaria siceraria*, and *Luffa acutangula* fruit was evaluated in Long Evans female rat against alloxan monohydrate. After 12 h, all the extracts (200 mg/kg i.p.) reduced fasting blood glucose level by 67.38, 65.39, and 51.10%, respectively. Reduced glycogen content (75.32%) of the diabetic rat was attenuated by treatment with *Luffa acutangula* (149.35%) ethanolic extract (Sharmin et al., 2013).

In another study, dose dependant glucose-lowering effect of methanolic fruit extract was observed in a Swiss albino mice (Juma et al., 2013).

Furthermore, Mohan Raj et al. (2012) examined the antidiabetic effect of the lyophilized ethanolic fruit extract (50%) against streptozotocin-induced diabetic Wistar rats. Two different doses, i.e., 200 and 400 mg/kg were administered orally for 21 days and different biochemical parameters were evaluated. Blood glucose level was significantly reduced in a dose-dependent manner along with decreased serum levels of SGPT, SGOT, and ALP were observed. No significant changes were observed in body weight and food intake of the animal at the end of the study (Mohan Raj et al., 2012).

In another study, Pimple et al. (2011) investigated the hypoglycemic effect of an aqueous and methanolic fruit extract against streptozotocin-induced diabetes in Swiss albino mice.

After 21 days, decreased levels of fasting serum glucose, glycosylated Hb, ALT, and AST along with improved liver glycogen levels were observed which thought to be contributed to attenuate hyperglycemic condition in mice (Pimple et al., 2011).

Patil et al. (2010) compared the antidiabetic potential of leaves of *Grewia asiatica*, bark of *Bombax ceiba*, and fruits of *Luffa acutangula* against alloxan-induced diabetic Wistar rats. Ether, chloroform, ethanol, and aqueous extracts (200 mg/kg b.w.) of each plant were administered orally and compared with standard glibenclamide (10 mg/kg b.w.). The results of the acute study showed significant blood glucose reduction from chloroform and alcoholic fruit extract (Patil et al., 2010).

Quanico et al. (2008) compared the hypoglycemic activity of methanol extract of *Bixa orellana*, *Kyllinga monocephala*, and *Luffa acutangula* leaves in an OGTT in Swiss Webster mice. *Luffa acutangula* extract showed significant glucose lowering activity when administered after 15 min of glucose load in the rat (Quanico et al., 2008).

In 2014, Singh et al. (2014) studied hydro-alcoholic extract of *Luffa acutangula* and *Madhuca longifolia* against alloxan-induced diabetic Wistar rat and observed a significant reduction in glucose level in the diabetic rat (Singh et al., 2014).

The results obtained under antidiabetic activity supports the traditional use of *Luffa acutangula* as an antidiabetic agent. Although it possess antidiabetic action, the effect in human is still unsatisfactory as in human diabetes treatment and should be studied extensively.

### Antihyperlipidemic Activity

Sharmin et al. (2013) compared the lipid-lowering effect of ethanolic extract (95%) of *Cucumis sativus*, *Lagenaria siceraria*, and *Luffa acutangula* fruit against alloxan monohydrate in Long Evans female rats. Extract significantly reduced total cholesterol (TC), TG, and low-density lipoprotein (LDL) levels by 38.38, 79.64, and 85.66%, respectively, in the serum of rat (Sharmin et al., 2013).

In another study, Pimple et al. (2011) established antihyperlipidemic activity of an aqueous and methanolic extract of fruit (200 and 400 mg/kg) in streptozotocin-induced diabetic mice. Oral administration of extract for 21 days, significantly (*P* < 0.05) increased levels of high-density lipoprotein and reduced serum TC, TGs, low-density lipoprotein, very low-density lipoprotein (Pimple et al., 2011). Only few *in vivo* studies are available for antihyperlipidemic effect of *Luffa acutangula*. Moreover, further preclinical and clinical studies should be performed to check its profound effect on blood lipid levels.

### Anticancer Activity

Anti-cancer potential of a methanolic and aqueous extract of fruit was studied in Dalton’s Lymphoma Ascites (DLA) cell induced solid tumor model. In the study, Swiss albino mice received two doses (200 and 400 mg/kg, oral) of each extract along with DLA cells. Development of solid tumor in mice was significantly diminished on treatment with both extracts (Dashora and Chauhan, 2015).

Furthermore, growth inhibitory effect of ethanolic extract of leaf was investigated on human lung cancer cell line (NCI-H460). The IC_50_ value was found to be at 20 μg/ml in MTT assay while cell lines showed high DCF fluorescence and significantly increased mitochondrial depolarization indicating anticancer activity of the extract (Vanajothi et al., 2012). However, not sufficient studies were undertaken to prove anticancer activity of the plant, due to which it is quite early to come to any conclusion. *In vitro* and *in vivo* anticancer studies are recommended to prove anticancer efficacy of plant.

### Analgesic and Anti-inflammatory Activity

Anti-inflammatory effect of ethyl acetate and ethanol extracts of dried leaves was compared by Iyyamperumal et al. (2013) using carrageenan-induced hind paw edema and cotton pellet granuloma models. In acute carrageenan induced model, ethanolic extract showed 67.6% and 72.5% edema inhibition, while ethyl acetate showed 62.5% and 65% inhibition at the doses of 250 and 500 mg/kg, respectively. Ethanolic extract showed 43.5% and 56.9% edema inhibition while ethyl acetate showed 36.5% and 52% inhibition at the doses of 250 and 500 mg/kg, respectively, in chronic cotton pellet granuloma model (Iyyamperumal et al., 2013).

Gill et al. (2011) investigated analgesic and anti-inflammatory activity of ethanolic seed extract in the albino rats. Anti-inflammatory activity (100, 200, and 300 mg/kg, oral) was evaluated by carrageenan-induced paw edema and analgesic activity (200 and 400 mg/kg, oral) by tail flick and tail immersion methods. Significant anti-inflammatory activity at the dose of 300 mg/kg while analgesic activity at the dose of 400 mg/kg was shown by seed extract (Gill et al., 2011). Taken together these reports support the traditional use of *Luffa acutangula* as pain relieving agent but the results are till unconvincing as humans are not involved in those studies. Hence, plant should be studied extensively as analgesic and anti-inflammatory agent.

### Antibacterial Activity

Recently several studies have been performed to highlight the ability of various extracts of *Luffa acutangula* to prevent growth of microbial strains. Silver nano-particles prepared from an aqueous extract of leaves exert significant antimicrobial activity against Gram-positive bacteria than Gram-negative bacteria (Moideen and Prabha, 2014).

*In vitro* antimicrobial potential of methanolic and aqueous extracts of fruit, seed, leaves, and root was examined by Jadhav and Chavan (2013) using well diffusion assay. The maximum zone of inhibition was shown by methanolic extract with few exceptions. The methanolic extract of all parts showed potent inhibitory action against *E. coli* and *Staphylococcus aureus* while that of fruit and leaves showed significant inhibition against *Klebsiella pneumonia*. Also, inhibition of *Fusarium* sp. was more in methanolic extracts of fruit and root when compared with other parts. Both extracts of leaves were effective against *Aspergillus niger*. The overall result of the study indicated that antimicrobial activity of different parts was solvent dependent due to phyto-constituents present in it (Jadhav and Chavan, 2013).

In another study, potent antimicrobial and antifungal activity was exhibited by fruit extract when compared with leaf extract. The area of inhibition was higher in *E. coli* than in *Staphylococcus aureus* and *Pseudomonas aeruginosa* species (Dandge et al., 2012).

Chloroform and aqueous extracts of the fruit were screened for their antimicrobial and antifungal potential. Antimicrobial activity was evaluated against Gram-positive bacteria (*Streptococcus aureus, Bacillus subtilis*) and Gram-negative bacteria (*Pseudomonas aeruginosa*, *Escherichia coli*) while antifungal activity was evaluated against *Candida albicans*, *Aspergillus niger*, *Aspergillus fumigates.* The chloroform extract exhibited more potent activity against Gram-negative bacteria and antifungal activity in MIC study (Torvi and Hunashal, 2012).

Furthermore, antibacterial activity of *n*-hexane, chloroform and ethyl acetate extracts of leaves were investigated by Bulbul et al. (2011) using disk diffusion method. *n*-Hexane extract exhibited most potent inhibitory activity followed by chloroform extract whereas ethyl acetate extract showed little or no activity (Bulbul et al., 2011).

The above data suggested that plant possess good antibacterial activity which supports its traditional use, but further research is needed to isolate bioactive compounds and understand their antibacterial mechanism.

### Immunomodulatory Activity

Ethanolic extract (100 and 200 mg/kg, p.o.) of fruit pericarp were investigated for immunomodulatory activity in Swiss albino mice. The evaluation of phagocytic index revealed that administration of ethanolic extract (200 mg/kg) in Indian ink intoxicated mice led to increase in phagocytosis to 0.028 ± 0.002 (*P* < 0.01). Also the % neutrophil adhesion in mice (200 mg/kg) was increased to 24.63 ± 0.87% which was more than standard drug Levamisol (23.58 ± 0.46%) (Kalasakar and Surana, 2014). Further investigations are needed to provide evidence for its immunomodulatory activity.

### CNS Depressant Activity

Misar et al. (2004) examined CNS depressant activity of ethanolic fruit extract (5 and 10 mg/kg, p.o.) in Swiss mice. The dose of the extract was safe up to 50 mg/kg treatment without any morbidity. CNS depressant activity was evaluated using behavioral changes, exploratory activity and barbiturates sleeping time animal models. The result of the study showed that CNS depressant activity of extract is dose dependant (Misar et al., 2004). More *in vitro* and *in vivo* studies should be performed to confirm the CNS depressant action of plant.

### Antiulcer Activity

Gastroprotective effect of dried fruit pulp extract (methanolic and aqueous) was investigated in NIDDM rat. Diabetes was induced by streptozotocin (65 mg/kg i.p.) along with nicotinamide (125 mg/kg, i.p.) and ulcer in the diabetic rat was induced by aspirin (200 mg/kg, p.o.). Increased cellular SOD and catalase level while restored mucosal glycoprotein levels were observed in gastric mucosa of rat treated with methanolic extract. The aqueous extract was less effective than methanolic extract in altering delayed healing of gastric ulcer in diabetic rats. In addition, methanolic extract exhibited dose-dependent glucose lowering and mucosal defensive action (Pimple et al., 2012).

## Toxicity Studies

The LD_50_ value of an aqueous and methanolic fruit extract was obtained at 4 g/kg body weight (Dashora and Chauhan, 2015). No mortality was observed for both extract during study period. The result indicated LD_50_ value for ethanolic extract at 500 mg/kg, while that of petroleum ether extract at 350 mg/kg (Ibrahim et al., 2014). In another study, the cytotoxic activity of ethanolic and pet ether extracts of aerial parts was evaluated in brine shrimp (*Artemia salina*) lethality model. Different concentrations of both extracts ranging from 1 to 500 μg/kg were used for the study and the LC_50_ value of ethanolic and pet ether extract was found to be at 32.8 ± 1.62 and 175.65 ± 10.80 μg/kg, respectively. The cytotoxic activity of *Luffa acutangula* was attributed to the presence of tannin in the extract (Rahman et al., 2014). Furthermore, ethanolic and ethyl acetate extract of leaves were investigated for acute oral toxicity as per OECD guideline no. 423. Both extracts were found to be safe at dose of 2,000 mg/kg (Iyyamperumal et al., 2013).

Arunachalam et al. (2012) examined for the safety of an ethanolic extract of whole plant using acute and chronic toxicity studies. The acute toxicity study was performed according to OCED guideline no. 423 where defined doses (2,000, 1,000, 500, 50, 5 mg/kg) were administered to Wistar rats and parameters like change in body weight, changes in skin and fur, motor pattern and behavior pattern of animals were observed for 14 days. No toxicity and deaths were observed at the dose of 2,000 mg/kg level, therefore 1/20th (100 mg/kg), 1/10th (200 mg/kg) and 1/5th (400 mg/kg) doses were selected for chronic toxicity study for 6 weeks. The hematological parameters (Hb concentration, clotting time, neutrophils, eosinophils, lymphocytes, monocytes, RBC, and WBC) evaluation of treated group showed no changes when compared to the control group. The biochemical parameters such as SGOT, SGPT, cholesterol, creatinine, urea, uric acid, protein, glucose and serum ALP also remained unchanged at the end of the study (Arunachalam et al., 2012).

Lyophilized ethanolic extract (50%) of fruit was investigated for its safety by Mohan Raj et al. (2012) using OECD-423 guideline. The lyophilized formulation was found to be safe up to dose of 2,000 mg/kg without any mortality (Mohan Raj et al., 2012).

In a study performed by Pimple et al. (2012), methanolic and aqueous extracts were safe in Swiss albino mice up to the dose of 2,000 mg/kg p.o. No autonomic or behavioral changes were detected during first 24 h. At the end of study no mortality was reported in animals even after 14 days of observation (Pimple et al., 2012).

The cytotoxicity of *n*-hexane, chloroform, and ethyl acetate extract of leaves were studied by Bulbul et al. (2011) using brine shrimp lethality bioassay with *n*-hexane extract of leaves showing significant LC_50_ value (20.40 μg/ml) when compared with chloroform (21.25 μg/ml) and ethyl acetate (23.09 μg/ml) (Bulbul et al., 2011).

Furthermore, abortifacient effect of fruit tea was evaluated by Fernandes et al. (2010) in pregnant Wistar female rats. On 15th gestational day, pregnant female rats were treated with *Luffa acutangula* fruit tea (10 ml/kg, p.o.) and the cesarean was done on the 21st day. No significant changes in body weight and no signs of maternal toxicity in the female rat were observed, but reduced fetus weight was reported. Since, weight is an important parameter in the fetus development, therefore, *Luffa acutangula* considered as fetoxic in nature (Fernandes et al., 2010).

In another study, acute toxicity of hydro-alcoholic (70%) extract of fruit was investigated using OECD guideline no. 425 in mice. Animals were administered with different doses (500, 750, 1,000, and 2,000 mg/kg) of hydro-alcoholic (70%) extract and observed for 72 h for clinical signs, symptoms, and mortality. Result depicted that no mortality was observed up to 10 g/kg dose level, even after 72 h (Jadhav et al., 2010).

Ulaganathan et al. (2010) evaluated ethanolic extract of leaves for acute toxicity study in Wistar rats. The extract was dissolved in Tween 80 solution and administered at the concentrations of 50, 100, 250, 500, 1,000, and >2,000 mg/kg by the oral route and the animals were observed for 72 h for any mortality or toxic symptoms. The result indicated that ethanolic extract of leaves did not show any toxic symptoms or mortality up to dose of 2,000 mg/kg (Ulaganathan et al., 2010).

Furthermore, ether, chloroform, ethanol, and aqueous fruit extracts were screened for safety using OECD guideline 423 and the study results exhibited 50% mortality with oral dose of 2,000 mg/kg (Patil et al., 2010).

## Conclusion And Future Perspectives

The current review documented existing information on the ethnobotanical uses, phytochemistry, pharmacology, and toxicology of *Luffa acutangula*. The amount of data gathered from different studies revealed that the plant is rich in many nutrients and vast biological active constituents.

Various modern pharmacological studies have been conducted to appraise the traditional uses of *Luffa acutangula* and research data obtained supported the traditional claims. Plant possess the potential multiple biological and therapeutic activities in the management of hepatoprotective, antidiabetic, antiulcer, anticancer, CNS depressant, fungistatic, analgesic, antimicrobial, immunomodulatory, etc., which can be deciphered by the presence of various constituents like RIPs, flavonoids, fatty acid, triterpenoids, and volatile components in it.

However, extensive investigations are required to formulate co-relations between the biological activities and chemical nature of the bioactive compounds deracinated from herb. Toxicity evaluation of plants is very important to understand their safety profile. To this end, no human studies have been performed to evaluate toxic effects of *Luffa acutangula*. The acute toxicity studies of the plant in preclinical models revealed fetotoxic nature as it reduces the weight of the fetus during pregnancy. However, further clinical evaluation must be performed to perceive the detailed effect of plant on human fetus. No other toxic effects were observed in preclinical studies indicating the safe use of plant.

From present compilation, it is observed that researchers need to establish the relationship between structure and function along with clinical studies on the efficacy of plants chemical components. Furthermore, validating the link between the traditional uses and therapeutic effects should be carried out further, and the toxicity of this plant also should be studied systematically. Well designed and strictly controlled clinical trials are also needed to validate safety and efficacy of dose before its recommendations for human consumption.

## Author Contributions

SB and PS conceived the review. PS drafted the manuscript. SB was involved in the editing process. Both the authors read and approved the final version of the manuscript.

## Conflict of Interest Statement

The authors declare that the research was conducted in the absence of any commercial or financial relationships that could be construed as a potential conflict of interest.
